# Rapid Growth and Spantaneous Expulsion of Hydatids from the Uterus

**Published:** 1853-03

**Authors:** Jas. Prentice

**Affiliations:** Fort Winnebago


ART. IV. *Rapid Growth and Spontaneous Expulsion of Hydatids from the Uterus, by* DR. JAS. PRENTICE.

I WAS called on the morning of the 19th January, '53, to see Mrs. C——, aged 19; found her with the usual symptoms dependent upon repeated hemorrhage, and having regular uterine contraction with hemorrhage. The history of her case, as near as I could learn, was as follows: She had menstruated regularly up to the month of October last, when the catamenia ceased, and the usual symptoms dependent upon gestation set in; and, for the last two months, she had noticed a rapid enlargement of her abdomen, and during that time she had repeated hemorrhage from the uterus, usually occurring at night; had felt occasional movements of the womb; had difficulty in passing urine; but had no pain simulating that of uterine contraction until the night previous to my being called.

Upon placing the hand upon the abdomen, the contour of the uterus could be distinctly felt through its parietes, the fundus being as high up as the umbilicus. An examination, per vaguum, revealed nothing except the descent of the uterus into the cavity of the pelvis. Ordered her to be kept quiet as possible, with her head low in bed, ℞ plumbi acetatis, gr. 3. Visited her again at noon; symptoms the same as in the morning, with the exception of a slight moderation of the hemorrhage. Continued the acetate of lead, alternating with tannic acid. During the after. noon, the uterine contractions increased in frequency and force, and towards evening the os incæ became dilated, and upon examination a soft pulpy mass presented, easily penetrated with the finger; detached portions soon began to pass at each contraction of the womb, and the hemorrhage became alarming. Gave ergot, and ordered gentle manipulation over the abdomen.

In the course of half an hour the whole tumor was expelled, and was composed of hydatids intermixed with a substance resembling coagulating lymph. The last portion that was expelled resembled in shape, color and tenacity that of healthy liver, and seemed to serve as a nucleus for the attachment of hydatids. The tumor I should judge must have weighed six or seven pounds, as I carefully removed the seperate portions as they escaped from the vagina and placed them in a vessel by themselves, and the clots of blood in another. The hemorrhage subsided. Applied a bandage to the patient as after child-birth, and left her for the night, after enjoining upon her the necessity of carefully maintaining the horizontal position.

20th. Found the patient comfortable, though very weak. Has since had slight fever, but is slowly convalescing.

One remarkable feature about the case is, the rapidity with which the hydatids were produced, for, judging from the history of the case, they could not have been more than four months in accumulating.

Fort Winnebago, Feb. 7th, 1853.

